# Multivitamin and Iron Supplementation to Prevent Periconceptional Anemia in Rural Tanzanian Women: A Randomized, Controlled Trial

**DOI:** 10.1371/journal.pone.0121552

**Published:** 2015-04-23

**Authors:** Nilupa S. Gunaratna, Honorati Masanja, Sigilbert Mrema, Francis Levira, Donna Spiegelman, Ellen Hertzmark, Naomi Saronga, Kahema Irema, Mary Shuma, Ester Elisaria, Wafaie Fawzi

**Affiliations:** 1 Department of Global Health and Population, Harvard T. H. Chan School of Public Health, Boston, Massachusetts, United States of America; 2 Ifakara Health Institute, Dar es Salaam, Tanzania; 3 Departments of Epidemiology, Biostatistics, and Nutrition, Harvard T. H. Chan School of Public Health, Boston, Massachusetts, United States of America; 4 Department of Epidemiology, Harvard T. H. Chan School of Public Health, Boston, Massachusetts, United States of America; 5 Muhimbili University of Health and Allied Sciences, Dar es Salaam, Tanzania; 6 Departments of Global Health and Population, Epidemiology, and Nutrition, Harvard T. H. Chan School of Public Health, Boston, Massachusetts, United States of America; Hunter College, UNITED STATES

## Abstract

**Objective:**

Women’s nutritional status during conception and early pregnancy can influence maternal and infant outcomes. This study examined the efficacy of pre-pregnancy supplementation with iron and multivitamins to reduce the prevalence of anemia during the periconceptional period among rural Tanzanian women and adolescent girls.

**Design:**

A double-blind, randomized controlled trial was conducted in which participants were individually randomized to receive daily oral supplements of folic acid alone, folic acid and iron, or folic acid, iron, and vitamins A, B-complex, C, and E at approximately single recommended dietary allowance (RDA) doses for six months.

**Setting:**

Rural Rufiji District, Tanzania.

**Subjects:**

Non-pregnant women and adolescent girls aged 15–29 years (n = 802).

**Results:**

The study arms were comparable in demographic and socioeconomic characteristics, food security, nutritional status, pregnancy history, and compliance with the regimen (p>0.05). In total, 561 participants (70%) completed the study and were included in the intention-to-treat analysis. Hemoglobin levels were not different across treatments (median: 11.1 g/dL, Q1-Q3: 10.0–12.4 g/dL, p = 0.65). However, compared with the folic acid arm (28%), there was a significant reduction in the risk of hypochromic microcytic anemia in the folic acid and iron arm (17%, RR: 0.61, 95% CI: 0.42–0.90, p = 0.01) and the folic acid, iron, and multivitamin arm (19%, RR: 0.66, 95% CI: 0.45–0.96, p = 0.03). Inverse probability of treatment weighting (IPTW) to adjust for potential selection bias due to loss to follow-up did not materially change these results. The effect of the regimens was not modified by frequency of household meat consumption, baseline underweight status, parity, breastfeeding status, or level of compliance (in all cases, p for interaction>0.2).

**Conclusions:**

Daily oral supplementation with iron and folic acid among women and adolescents prior to pregnancy reduces risk of anemia. The potential benefits of supplementation on the risk of periconceptional anemia and adverse pregnancy outcomes warrant investigation in larger studies.

**Trial Registration:**

ClinicalTrials.gov NCT01183572

## Introduction

Anemia during pregnancy has been associated with adverse outcomes including maternal and perinatal mortality, pre-term delivery, and low birth weight [1, 2]. While iron deficiency is considered the leading cause of anemia, other causes include deficiencies in micronutrients such as folate, vitamin B12, and vitamin A, infestations with parasites such as helminths or malaria, genetically-inherited hemoglobinopathies, HIV/AIDS, and chronic inflammation [3, 4]. Iron requirements increase over the course of pregnancy and, later in pregnancy, exceed amounts that could be absorbed even from an optimal diet [5]. Pregnant women must therefore enter pregnancy with sufficient iron stores to meet their full requirements. However, many women, especially in developing countries, enter pregnancy with iron as well as other nutrient deficiencies and consequently suffer from nutritional anemias due to food insecurity, suboptimal diets, increased morbidity, early initiation of childbearing, multiple pregnancies, short inter-pregnancy intervals, and prolonged lactation [6]. Nutrient availability is important around conception and during early pregnancy for key developmental processes including implantation, placental formation and function, embryogenesis, and fetal organogenesis [7]. For most women, these needs occur before they seek antenatal care. Nutrient supplementation of women of reproductive age may therefore be an effective strategy to improve nutritional status of at-risk women prior to and in preparation for pregnancy.

There is growing interest in the effect of maternal pre-pregnancy and periconceptional nutritional status on maternal and infant outcomes. The benefits of periconceptional folic acid supplementation for reducing the risk of neural tube defects has been well demonstrated through randomized, controlled studies [8] as well as evaluations of national fortification programs [9]. Preconceptional anemia among women of reproductive age in China was associated with fetal growth restriction and lower birth weight [10]. Preconceptional iron-deficiency anemia in particular had a strong negative association with birth weight, as did anemia related to deficiency of at least one of folate, vitamin B6, or vitamin B12. An intervention to provide weekly iron and folic acid supplements to women of reproductive age in Vietnam reduced anemia, iron deficiency, and iron-deficiency anemia in non-pregnant women with benefits that continued through the first and second trimesters of pregnancy; however, in the third trimester, the rates of these endpoints were comparable with those of women receiving daily iron-folic acid supplements with no preconceptional supplementation [11]. Using a shared protocol and similar target populations [12], a second study in the Philippines found that weekly iron-folic acid supplementation improved serum ferritin and hematocrit but not hemoglobin [13], while a third study in Cambodia found improved hemoglobin levels with increasing effectiveness among women of higher socioeconomic status [14]. Randomized studies on the efficacy of supplementation to improve periconceptional anemia, however, are lacking in Africa, where nutritional deficiencies are widespread, the burden of anemia is high among non-pregnant (48%) and pregnant (57%) women, and there is some evidence that these rates are increasing [15, 16].

In Tanzania, 40% of women of reproductive age are anemic, with significant variation among regions [17]. Eleven percent are underweight, with more undernutrition in rural than in urban areas. Among rural mothers, less than one in three consume iron-rich foods in a 24-hour period, and only 3% took iron supplements for 90 or more days during their last pregnancy. Meanwhile, 58% of girls initiate sexual activity by the age of 18 years, with the fertility rate at 5.4 children per woman and the median birth interval below three years [17]. While 98% of Tanzanian women seek antenatal care at some time during pregnancy, the median gestational age at the first antenatal visit is over five months [18]. We therefore conducted a randomized controlled trial of iron and multivitamin supplementation among non-pregnant women and adolescent girls in a rural district of Tanzania to determine the effect of supplementation on anemia during the periconceptional period.

## Methods

### Ethics statement

The study was approved by the Institutional Review Boards (IRBs) at the Harvard School of Public Health, Ifakara Health Institute, and National Institute for Medical Research (Tanzania) and registered in the United States Clinical Trials registry (ClinicalTrials.gov identifier NCT01183572). The Tanzania Food and Drug Authority approved the use of the regimens. All participants provided written informed consent themselves or through a legal guardian if under 18 years of age.

### Study design and intervention

A double-blind, randomized controlled trial of non-pregnant women and adolescent girls was conducted in Ikwiriri and Kibiti, two rural wards in Rufiji District, Pwani Region, Tanzania. Participants were recruited between October 2010 and June 2011 and randomly assigned to receive daily oral supplements of folic acid alone, folic acid and iron, or folic acid, iron, and vitamins A, B-complex, C, and E. All supplements were provided for six months in approximately single recommended dietary allowance (RDA) doses (Table 1) [19–21]. The three study supplements were manufactured by Tishcon Corporation (Salisbury, MD) and were indistinguishable in appearance and taste. The primary objective was to determine whether daily oral supplementation with folic acid and iron or with folic acid, iron, and multivitamins would reduce the risk of anemia when compared with supplementation with folic acid alone. Given its known benefits during the periconceptional period [8, 9], folic acid was provided as the comparison regimen rather than a placebo.

**Table 1:** Daily amounts of vitamins and iron provided by the study regimens.

		Regimen		
Nutrient	Amount	Folic acid only	Folic acid + iron	Folic acid, iron, multivitamins
Folic acid (mg)	0.4	X	X	X
Iron (mg)	30		X	X
Vitamin A (IU)	2500			X
Vitamin B1 (mg)	1.4			X
Vitamin B2 (mg)	1.4			X
Vitamin B6 (mg)	1.9			X
Vitamin B12 (mg)	2.6			X
Niacin (mg)	18			X
Vitamin C (mg)	70			X
Vitamin E (mg)	10			X

Women were eligible for the study if they were between 15 and 29 years of age, not pregnant, planning to remain in the study area for six months, and willing to provide written informed consent themselves or through a guardian if under 18 years of age. Women were excluded if they had amenorrhea, had given birth within the past six months, were already taking vitamin supplements, or had any severe illness requiring hospitalization during screening or enrollment. In total, 802 women and adolescent girls were enrolled. While the initial target sample size was 1800 participants, as this study was intended as a pilot to assess the feasibility of micronutrient supplementation in a rural population during the periconceptional period, the final sample size was based on the timeline and resources available, and this change was reported to the IRBs. Of the enrolled participants, 561 or a minimum of 183 per treatment arm completed the laboratory evaluation following six months of supplementation. In a pairwise comparison of a treatment arm to the arm providing folic acid alone, the study had 80% power at a 5% significance level to detect a 0.5 g/dL change in hemoglobin level using an independent samples t-test or a 29% reduction (absolute reduction of 14%) in the risk of anemia using a χ^2^ test of independence, assuming a standard deviation of 1.8 g/dL for hemoglobin and 50% prevalence of anemia in the folic acid arm.

### Recruitment and randomization

Potential participants were first screened for eligibility through an existing demographic surveillance system (DSS) in the two wards. Women were interviewed at their homes during a regularly scheduled DSS visit and those meeting the eligibility criteria were referred to their ward health clinic where their eligibility was confirmed, they received information about the study, and informed consent was obtained. All women who travelled to their ward health clinic were reimbursed for round-trip travel expenses regardless of whether they enrolled in the study. During household screening, women were initially considered not pregnant if they had had a menstrual period in the preceding four weeks; however, given that some women had delayed menstrual periods due to naturally longer cycles, lactation, or contraceptive use or had poor recall of their menstrual history, this criterion was revised for initial screening to having had a menstrual period in the preceding six weeks. During eligibility confirmation at ward clinics, women determined not to have had a menstrual period in the preceding four weeks were then given a urine pregnancy test to confirm absence of pregnancy.

Eligible, consenting participants were individually randomized in equal numbers to one of the three interventions (Table 1) and given their first bottle of regimen with instructions for use. Allocation was performed according to a computer-generated randomization sequence using blocks of size 15 created by a scientist not involved in data collection or analysis. At enrollment, research staff assigned each eligible person to the next numbered bottle at that site. At each subsequent visit, study supplements were dispensed in identical bottles labeled with the participant’s study identification number and prepared by pharmacists who had no contact with the participants. All participants and research staff were blinded to the intervention allocation. During randomization, data were also collected on participants’ demographic and socioeconomic characteristics, diet using a food frequency questionnaire, height, weight, and middle upper arm circumference (MUAC).

### Follow-up

Following randomization, study supplements were dispensed during monthly home visits, during which data were collected on compliance with the regimen, reasons for non-compliance, any reported symptoms since the last study visit, and menstrual history. Compliance was assessed through participants’ self-reporting and pill counts. The final study visit was conducted at the local ward clinic, where participants’ height, weight, and MUAC were measured and peripheral venous blood was collected by trained laboratory technicians using EDTA vacutainer tubes and transported to Mchukwi Mission Hospital in Mchukwi, Pwani Region, for determination of complete blood cell counts using a Sysmex KX-21N Automated Hematology Analyzer. Peripheral venous blood was also used to prepare a thin blood smear to type anemia and a thick blood smear that was read by two independent laboratory technicians to assess malaria parasitemia. A third reading was conducted as needed to resolve discrepant readings. Though originally planned, these laboratory assessments could not be obtained on the study participants at baseline. Information was obtained from relatives or neighbors if a participant was not available for a scheduled visit at home or at the clinic, and all efforts were made to reach participants who traveled within the study area for family reasons or for work including seasonal farm labor.

### Outcome measures

Hemoglobin concentration (Hb), mean corpuscular volume (MCV), red cell distribution width (RDW, measured as the coefficient of variation or CV of the MCV x 100 [22]), and mean corpuscular hemoglobin (MCH) were measured by complete blood cell count. Anemia was defined as Hb<11 g/dL, severe anemia as Hb<8.5 g/dL, microcytosis as MCV<80 fL, and hypochromia as MCH<27 pg [22, 23]. Hypochromic microcytic anemia was defined as Hb<11 g/dL, MCV<80 fL, and MCH<27 pg. Normocytic anemia was identified as Hb<11 g/dL and 80≤MCV≤100 fL. Malaria infection was identified by presence of *Plasmodium* parasites observed by light microscopy in a thick peripheral blood smear. Compliance with the regimen was calculated as the number of tablets taken from all returned regimen bottles divided by the total number of days the participant used those bottles.

### Statistical analysis

Analyses were based on the intention-to-treat principle and included all randomized participants. Distributions of quantitative outcomes were summarized using the median, the first quartile (Q1), and the third quartile (Q3) of the observed distributions. Differences in baseline measures among the treatment arms were assessed with χ^2^ tests of independence for categorical variables and Kruskal-Wallis tests for continuous variables. Pairwise comparisons of outcomes in each treatment arm versus the folic acid arm were made using χ^2^ tests of independence for categorical variables and Wilcoxon rank-sum tests for continuous variables. The relative risk (RR) of an outcome was calculated as the proportion of participants experiencing the outcome in a given treatment arm divided by the proportion of participants experiencing the outcome in the folic acid arm, and 95% confidence intervals (CI) for relative risks were calculated using a standard formula [24]. To adjust for any potential selection bias due to loss to follow-up, the main analysis of differences in outcomes between the two treatment arms and the folic acid arm was repeated using inverse probability of treatment weighting (IPTW) [25]. Weights were calculated as the inverse of predicted values from a logistic regression of the following categorical variables on loss to follow-up: infrequent (less than once per week) household meat consumption, underweight status (body mass index [BMI] < 18.5 kg/m^2^) at baseline, high parity (two or more previous pregnancies) at baseline, breastfeeding status at baseline, ever reporting side effects (e.g., nausea, vomiting) during the intervention, ever reporting a feeling of having more energy or strength during the intervention, ever reporting better ability to concentrate during the intervention, ever reporting better ability to work during the intervention, and low compliance (supplements taken on less than 80% of days during the intervention). These factors were chosen because they were plausible risk factors for anemia or possible consequences of supplementation. For all outcomes, effects of the folic acid and iron and the folic acid, iron, and multivitamin treatments compared with the folic acid treatment were estimated using weighted log-binomial models with empirical standard errors [26]. Modification of the regimen effect on hypochromic microcytic anemia by infrequent household meat consumption, underweight status at baseline, high parity at baseline, breastfeeding status at baseline, and low compliance was assessed using the Breslow-Day test [24]. All P-values were two-sided. SAS version 9.2 (SAS Institute Inc., Cary, NC) was used for all analyses.

## Results

### Study population

**Fig 1:**
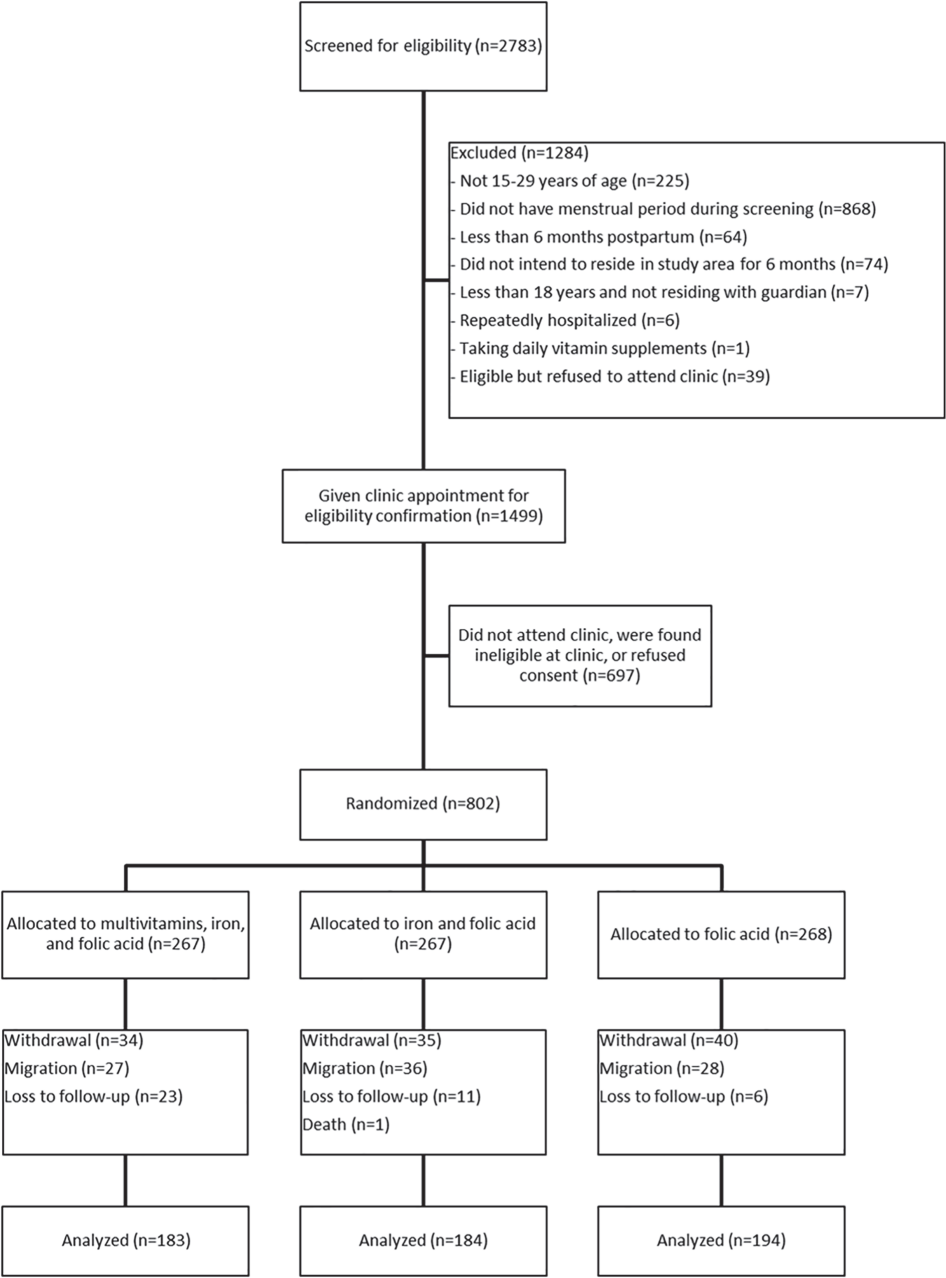
Flow of study participants.

Of the 2783 women screened for eligibility through the DSS, 1499 (54%) were referred to a clinic for eligibility confirmation and randomization (Fig 1). The primary reason for exclusion was the lack of a menstrual period during screening (n = 868). Relaxing the criterion from having had a menstrual period in the preceding four weeks to having had a menstrual period in the preceding six weeks allowed more women to be referred to their ward health clinics for further screening with the use of a urine pregnancy test if necessary. Use of nutritional supplements outside of pregnancy was very rare in this population, accounting for only 0.1% of exclusions through the DSS.

Of the women referred, 802 (54%) traveled to the clinics and were confirmed as eligible, consented, and randomized to one of the three study arms (Fig 1). Seventy percent (n = 561) completed the six-month intervention and underwent laboratory assessments at the endpoint, while 14% (n = 109) withdrew, the reasons for which included symptoms that may (e.g., nausea) or may not (e.g., skin rash) have been related to the intervention, pressure from relatives to discontinue, or new pregnancy. Eleven percent (n = 91) migrated out of the study area, reflecting the mobility of this population. Specific reasons for migration included a change in marital status (i.e., marriage or divorce), movement to live with relatives, or movement to a more urban area to seek a new livelihood. Five percent (n = 40) were lost to follow-up, some of whom moved temporarily for seasonal farming activities. One woman died from severe malaria prior to completion of the study. Recruitment and follow-up extended from October 2010 to December 2011, when all follow-up was completed.

The study arms were comparable with respect to demographic and socioeconomic characteristics, food security (as assessed by household per capita food expenditure and meat consumption), nutritional status (as assessed by BMI), and pregnancy history (p>0.05, Table 2). Study participants were young women aged 21 years on average. One in four was under 18 years. Thirty-seven percent were currently married, 57% had been previously pregnant, and 24% were breastfeeding at the time of randomization. Only a quarter of women had education beyond the primary level, and 18% had no formal education. The majority of participants were housewives, worked on the family farm, or were unemployed. Poverty was high in this population, with two-thirds of participants living in homes with mud floors and only 11% with access to electricity. Household food expenditures were low, and 42% of participants stated that they did not regularly purchase food, reflecting the high reliance on subsistence agriculture in this population. The majority of households consumed meat less than once per month. Seventeen percent of participants were underweight (BMI<18.5 kg/m^2^) and 14% were overweight (BMI≥25 and <30 kg/m^2^) or obese (BMI≥30 kg/m^2^). Compliance with the regimen did not differ by study arm, with a median compliance of 82% in the folic acid arm, compared with 84% in the folic acid and iron arm (p = 0.40) and 83% in the folic acid, iron, and multivitamin arm (p = 0.67).

**Table 2:** Basic characteristics of the study population at recruitment (n = 802).

	Regimen			P-value
	Folic acid (n = 268)	Folic acid + iron (n = 267)	Folic acid, iron, multivitamins (n = 267)	
Age (years)	21.2(4.5)	21.4(4.4)	20.5(4.2)	0.06
Married, %	37	41	33	0.14
Education				0.67
none, %	18	18	19	
any primary, %	54	54	57	
any secondary or above, %	28	28	23	
Employment				0.59
work outside home, %	15	20	15	
farming, %	32	31	30	
unemployed, %	36	36	39	
student or other, %	16	13	16	
Metal roof, %	63	58	53	0.07
Concrete or improved floor, %	33	33	30	0.79
Working electricity, %	13	9	10	0.31
Daily household per capita food expenditure (TZS)				0.22
0, %	45	38	44	
<1000, %	40	48	45	
> = 1000, %	15	14	11	
Household meat consumption				0.67
never, %	26	24	24	
less than 1/month, %	27	32	30	
1–3/month, %	32	26	29	
1+/week, %	15	18	17	
Body mass index (BMI, kg/m^2^)	21.6(4.1)	21.7(3.7)	21.2(3.4)	0.38
Previous pregnancies				0.15
0, %	45	38	46	
1, %	21	20	23	
2+, %	34	41	31	
Lactating, %	21	27	25	0.30

Values are means (SD) or percentages. TZS: Tanzanian Shilling. US$1 ≈ 1500 TZS during recruitment. P-values are calculated from χ2 tests of independence for categorical variables and Kruskal-Wallis tests for continuous variables.

### Anemia and malaria

Hemoglobin levels were not different in the three arms following the six-month intervention, with median values of 10.9 g/dL (Q1-Q3: 9.9–12.4) in the folic acid arm, compared with 11.1 g/dL (Q1-Q3: 10.0–12.4, p = 0.64) in the folic acid and iron arm and 11.4 g/dL (Q1-Q3: 10.0–12.4, p = 0.36) in the folic acid, iron, and multivitamin arm. Nearly half of women were anemic and 5% were severely anemic despite six months of supplementation, with no significant differences among the study arms (Table 3). Compared with the folic acid regimen (42%), the folic acid and iron regimen significantly reduced the risk of microcytosis (30%, RR: 0.72, 95% CI: 0.54–0.94, p = 0.02), and the folic acid, iron, and multivitamin regimen had a similar effect, though the result was not statistically significant (34%, RR: 0.81, 95% CI: 0.62–1.05, p = 0.12). Compared with the folic acid regimen (28%), the folic acid and iron regimen (17%, RR: 0.61, 95% CI: 0.42–0.90, p = 0.01) and the folic acid, iron, and multivitamin regimen (19%, RR: 0.66, 95% CI: 0.45–0.96, p = 0.03) significantly reduced the risk of hypochromic microcytic anemia. Pooling the iron-containing arms, iron supplementation with or without micronutrients reduced the risk of hypochromic microcytic anemia by 37% (95% CI: 13–54%, p = 0.005). Red cell distribution width was marginally reduced in the folic acid and iron (median CV: 13.5, Q1-Q3: 12.8–14.3, p = 0.08) and in the folic acid, iron, and multivitamin (median CV: 13.4, Q1-Q3: 12.6–14.3, p = 0.09) arms, compared with the folic acid arm (median CV: 13.7, Q1-Q3: 12.8–14.8). The study arms did not differ in risk of normocytic anemia or malaria infection following the intervention. IPTW to adjust for potential selection bias due to loss to follow-up did not materially change these results. The effect of the regimens on hypochromic microcytic anemia was not modified by infrequent household meat consumption; baseline underweight status, high parity, or breastfeeding status; or level of compliance (p>0.2).

**Table 3:** Effect of six months of supplementation with iron and multivitamins on risk of anemia and malaria.

	Regimen								
	Folic acid (n = 194)	Folic acid + iron (n = 184)				Folic acid, iron, multivitamins (n = 183)			
Outcome	%	%	RR	(95% CI)	P-value	%	RR	(95% CI)	P-value
Anemia (Hb<11 g/dL)	50	46	0.91	(0.74,1.13)	0.40	43	0.86	(0.69,1.07)	0.18
Severe anemia (Hb<8.5 g/dL)	6	5	0.86	(0.37,2.03)	0.74	5	0.87	(0.37,2.04)	0.74
Microcytosis	42	30	0.72	(0.54,0.94)	0.02	34	0.81	(0.62,1.05)	0.12
Hypochromia	73	67	0.92	(0.80,1.05)	0.22	64	0.89	(0.77,1.02)	0.09
Hypochromic microcytic anemia	28	17	0.61	(0.42,0.90)	0.01	19	0.66	(0.45,0.96)	0.03
Normocytic anemia	22	28	1.28	(0.90,1.83)	0.17	24	1.09	(0.75,1.58)	0.67
Malaria infection	4	3	0.85	(0.27,2.74)	0.79	6	1.69	(0.63,4.53)	0.29

## Discussion

Growing evidence on the importance of maternal nutritional status during conception and early pregnancy suggests that interventions to improve periconceptional nutritional status could reduce the risk of adverse maternal and perinatal outcomes. To the authors’ best knowledge, this is the first community-based randomized, controlled trial to provide nutrient supplementation to improve periconceptional anemia status in Sub-Saharan Africa. We found that daily oral supplementation with iron and folic acid prior to conception reduced the risk of hypochromic microcytic anemia by 37% in women and adolescent girls when compared with supplementation with folic acid alone. Iron deficiency is the primary cause of hypochromic microcytic anemia [27], and the high prevalence of this form of anemia and the population’s responsiveness to iron supplementation suggest that iron deficiency is a significant problem and supplementation would be beneficial.

Iron plays important roles during conception, placental formation, and early fetal development [7], and deficiency during this period can affect perinatal and infant outcomes directly through its influence on these processes [6, 28–30] or indirectly through effects on anemia and iron status later in pregnancy [10, 31, 32] or postpartum [33]. In Tanzania, iron and folic acid are provided during pregnancy through the health system; however, women often delay initiation of antenatal care [17, 18] and use of these supplements can be low [34]. The key periods of periconception and early pregnancy are therefore missed and the duration of subsequent supplementation may be insufficient to correct nutritional deficiencies or alleviate anemia. Pre-pregnancy iron and folic acid supplementation may therefore be efficacious in improving periconceptional anemia status and consequently maternal and infant outcomes. In this study, inclusion of multivitamins did not reduce the risk of anemia beyond the reduction from supplementation with iron and folic acid alone. However, statistical power may have been limited in this pilot study, and the potential effect of multivitamin supplementation on the risks of periconceptional anemia and adverse perinatal outcomes warrants further investigation in larger studies, particularly in Sub-Saharan Africa, where many women enter pregnancy with multiple nutrient deficiencies.

The trending but statistically non-significant findings for hemoglobin and anemia may have resulted from the effect of supplementation on cases of microcytic anemia being obscured by the lack of effect on normocytic cases. A larger study with more statistical power may detect overall effects on hemoglobin and anemia, as has been observed in other iron and folic acid supplementation studies [35, 36]. Further, folic acid supplementation alone may reduce anemia [32], reducing the observed differences between the treatment arms and the folic acid arm following supplementation. The lack of baseline data does not allow examination of whether hemoglobin levels increased or the risk of anemia decreased in the folic acid arm after supplementation. The lack of a baseline also prevents correction for baseline status in analyses of treatment effects; however, due to randomization, systematic differences in baseline status among the study arms are unlikely and the lack of correction for baseline status is consequently not expected to influence the results. Comparison of participant characteristics at baseline further found no differences among the study arms.

The use of hypochromic microcytic anemia as an indirect marker of iron deficiency anemia has limitations, given that hypochromic microcytic anemia has other causes such as hemoglobinopathies, in particular thalassemias. In Tanzania, α^0^ and β thalassemias are rare, while the prevalence of α^+^ thalassemia heterozygosity is approximately 2% [37]. While they can influence study outcomes, these genetic disorders are expected to be equally prevalent in each study arm due to randomization; therefore, they are not expected to influence the results.

Another study limitation is the potential bias arising from eligible women who elected not to travel to their local health clinics for study enrollment. While reimbursement was provided for round-trip travel expenses to encourage and facilitate enrollment of women with limited resources or those who lived further from the clinics, this reimbursement was only provided upon arrival at the clinic. Women who lacked cash for the initial journey or who had limited access to transportation in the rural and dispersed study area may have been less likely to enroll in the study. Women engaged in work or schooling outside the home or who had heavy responsibilities at home or on the farm including care for children or other family members may also have been less likely to travel for enrollment. Some of these women, particularly poorer women and those living in more remote or marginal areas, may have been at higher risk of anemia and nutritional deficiencies, indicating the need for strategies for difficult-to-reach populations.

Given high fertility and the nutritional demands of repeated pregnancies and prolonged periods of lactation, interventions, programs, and services aimed at improving nutritional status among adolescents before they initiate childbearing may be particularly effective in improving maternal and child outcomes. Improvement in a girl’s stature prior to childbearing can have positive intergenerational consequences [38, 39], and reducing anemia in adolescent girls and non-pregnant women would also have benefits beyond those related to future pregnancy, including increased work capacity and productivity, cognitive development, and resistance to infection, all of which have economic implications [40, 41]. Correcting iron and other nutritional deficiencies can also have positive effects on mental health, related to or separate from pregnancy [42, 43]. However, reaching non-pregnant women and adolescent girls remains a challenge, and more work is needed on effective entry points such as schools before girls complete or discontinue their education; events surrounding key life stages such as marriage; or health system interventions or programs such as visits by community health workers or wellness visits at health facilities based on age [44].

While daily pre-pregnancy supplementation of women and adolescent girls may be efficacious in reducing the risk of anemia and other nutritional deficiencies, it poses significant challenges for sustainable delivery and compliance. Weekly supplementation can be an effective alternative [45, 46]. However, a meta-analysis of daily or intermittent iron supplementation to reduce the risk of iron deficiency anemia concluded that neither is likely to be effective unless programs or interventions have good supervision and good compliance, and weekly supplementation may be disadvantageous when the baseline anemia prevalence is high [47]. Interventions providing weekly iron and folic acid supplements to women of reproductive age in Vietnam, the Philippines, and Cambodia were successful in improving anemia and iron status using social marketing, community mobilization, and a government-industry partnership to promote the approach [11, 13, 14].

Other delivery methods, such as micronutrient powders administered daily or on a flexible schedule, may have greater acceptance and compliance and should be investigated [48]. Community mobilization and modes of delivery other than supplement pills can be important in areas, such as this study’s location, where stigma against antiretroviral therapy (ART) for HIV infection is prevalent [49] and family or community members could confuse regular use of supplement pills with ART usage. Participants in this study were highly mobile, and flexible administration could also be useful as young women and adolescent girls leave school, migrate to urban areas, engage in work outside the home, marry, divorce, or travel to visit relatives, give birth, or engage in agricultural or other seasonal or short-term labor.

Intermittent supplementation may also be an effective option to prevent anemia in populations with a high burden of infectious diseases. The safety of iron supplements for individuals with malaria, HIV, and hepatitis B and C has been debated as iron could worsen infection [50–53]. However, these and other diseases, in particular malaria [54] and HIV [55], also cause anemia and are significant public health concerns in Tanzania. While iron supplementation did not increase the risk of malaria in this study, monitoring for potential harm is indicated especially in areas with high malaria prevelance, and intermittent supplementation may be an effective way to prevent anemia without providing excess iron to stimulate pathogens or parasites in endemic areas.

## Supporting Information

S1 CONSORT Checklist CONSORT Checklist. (PDF)

S1 Protocol Study protocol. (PDF)
