# Supplementary material for: Multivitamin and Iron Supplementation to Prevent Periconceptional Anemia in Rural Tanzanian Women: A Randomized, Controlled Trial
Source: PLoS One. 2015 Apr 23;10(4):e0121552. doi: 10.1371/journal.pone.0121552 (PMC4408096; doi:10.1371/journal.pone.0121552)
Supplement: S1 Protocol — (PDF) [file pone.0121552.s002.pdf]

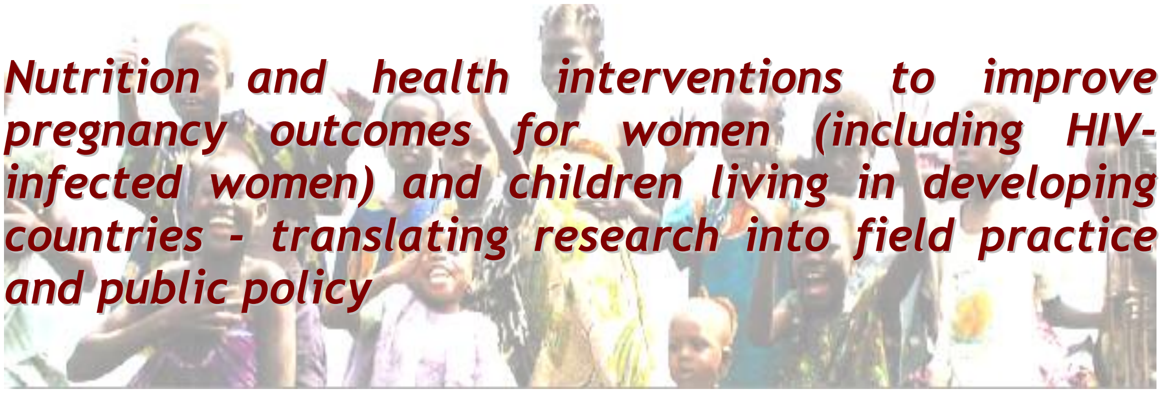

# Nutrition and health interventions to improve pregnancy outcomes for women (including HIV-infected women) and children living in developing countries - translating research into field practice and public policy

## *Trial of Pre-Pregnancy Supplements*

**A Collaborative Project between the Ifakara Health Institute, Ifakara and Rufiji, Tanzania, and the Harvard School of Public Health, Boston, MA**

**Harvard School of Public Health**
**Wafaie Fawzi, MD, DrPH**

**Ifakara Health Institute**
**Honorati Masanja, PhD**

Version date: September 8, 2010

## Background

In sub-Saharan Africa, millions of women risk death every year to give life to a newborn child. These women are also at high risk of delivering a child that experienced poor growth in the womb and that may thus develop poorly or may even die soon after birth. One important determinant of this dilemma of pregnant women is poor antenatal care. In Tanzania, a country that is among the 15 poorest countries in the world, 6 women die of pregnancy-related causes for every 1,000 births. One out of every 6 children is born with a low weight (< 2500gms) at birth and 68 out of every 1,000 live births die in the first year of life.

Periconceptional Maternal Nutritional Status: Pre-pregnancy low body mass index and short stature are known risk factors for adverse pregnancy outcomes. The benefit of periconceptional folate on preventing congenital anomalies has been established in randomized trials; however, the role of other nutrients needs to be examined further. First, improving nutritional status may be important to cover the period of placental formation and growth which is key for laying the foundation for improved placental immunity, and preventing placental malaria (and other infections), a major cause of adverse maternal and fetal outcomes. Second, anemia is strongly associated with adverse perinatal outcomes including maternal mortality and low birthweight. It causes circulatory decompensation and increased cardiac output, which coupled with the added stress of labor and blood loss during delivery, leads to circulatory shock and frequently maternal death. It is also related to postpartum anemia and depression. Conventionally, iron supplements are provided to women when they are pregnant. However, many women enter pregnancy with little or no iron reserves due to poor diet, closely spaced pregnancies, blood loss by postpartum hemorrhage, and prolonged periods of lactation. Therefore, it is very difficult to replenish iron stores once the pregnancy is in progress. Additionally, iron deficiency is responsible for only about half of the anemia burden in pregnancy, with other micronutrient deficiencies playing a major role. Third, periconceptional vitamin A supplementation may be important for maternal and fetal survival as well. Supplementation of women during childbearing years reduced pregnancy-related mortality by about 50% in undernourished women in a large trial from rural Nepal (n~45,000); however, the effects of vitamin A supplementation need to be confirmed in African settings with high burden of malaria and HIV infection. Fourth, free radicals generated during conception, fertilization, and early pregnancy lead to oxidative stress, degeneration of the syncytiotrophoblasts and early pregnancy failure. Early pregnancy loss in the form of first trimester and early second trimester abortions are not amenable to interventions through conventional antenatal care – few women in Tanzania complete an Antenatal visit within the first 12 weeks of pregnancy. Therefore contact with the health system during pregnancy is too late to prevent early abortions and pre-pregnancy supplementation will be a practical population intervention to tackle poor pregnancy outcomes especially early in pregnancy.

## Study Objectives and Aims:

1) To determine the predictors of poor compliance to multivitamin supplements among non-pregnant Tanzanian women. .

2) To determine whether daily oral supplements of multivitamins (including vitamins B-complex, C and E) along with Iron and folic acid given to non-pregnant women results in lower prevalence of anemia in preparation for pregnancy when compared to folic acid alone.

3) To develop a model to incorporate the effective use of multivitamin supplements during periconceptional care with a high degree of compliance in Tanzania that is sustainable and can be replicated in other low-income settings.

## Study Methods:

### *Study Site:*
Rufiji District of Coast Region of Tanzania

### *Study duration:*
From March 2010 to March 2011

### *Target Population:*
The target population will be women between the ages of from 15 and 29 years residing in the two townships of Kibiti and Ikwiriri of Rufuji. These areas have been under the Rufiji Health Demographic Surveillance System (RHDSS) of the Ifakara Health Institute for over a decade. Our research partners at the Ifakara Health Institute conduct health surveillance surveys in this area 3-4 times a year and therefore have a major presence and rapport here. Surveys conducted in these two townships in the past years indicate that at least 5600 girls and women in this age group reside in these two townships.

### *Planned Procedures:*
Our research staff will undergo a thorough 6-week training course on interviewing, counseling, and data collection techniques before starting this work. They will also receive training during this period on phlebotomy and laboratory investigations to be performed as part of the study. All work performed by research staff will be supervised by study supervisors and by Drs Honorati Masanja, and Wafaie Fawzi. These activities will be rigorously standardized, both the equipment used and the specific actions of the research staff.

### *Enrollment:*
As the first process as part of the study, we will undertake a door-to-door survey of all households that are located in the two townships of Kibiti and Ikwiriri of Rufiji in Tanzania. All women and girls aged between 15 and 29 years will be identified during this survey. This survey will be coordinated by the Rufiji Demographic Health Surveillance network in such a way that it coincides, at least in part, with the routine

RDHS activities and does not entail an additional visit inconveniencing the population. Eligibility for enrolment will be determined based on the following inclusion and exclusion criteria:

Inclusion criteria:

1. Girls aged > =15 years and <= 29 years
2. Have not missed a menstrual period during recruitment (no amenorrhea)
3. Has not been pregnant or given birth within the last 6 months
4. Intend to stay in the study area for at least 6 months after enrollment
5. Have provided written informed consent

Exclusion criteria:

1. Amenorrhea or confirmed pregnancy at screening or enrollment.
2. Has given birth within 6 months
3. Already taking long-term vitamin supplementation.
4. Any severe illness requiring hospitalization at screening or enrollment (women who have been deemed to have recovered will be eligible once they return home).

Screened prospective participants will be informed in detail about the study if eligible. Baseline information will be collected from eligible participants who have provided informed written consent and will then be randomized to receive either multivitamins or a taste, color and odor matched placebo. In households where more than one individual is eligible, the youngest individual will be enrolled. Experienced and well-trained research workers will seek consent for the study at the homes of participants. These study personnel are recruited from the same areas that they survey and will be trained by a team of researchers who have been conducting regular home visit based DSS in Rufiji for over a decade. They therefore enjoy a unique rapport with the population in the study area.

***Sample size:***

1800 Individuals will be enrolled into the trial, 600 to each limb (placebo, intervention 1 and intervention 2). We estimated that we would need 564 women in each group to be able to detect a 20% reduction (absolute reduction by 10%) in the prevalence of anemia (Hb < 12 gm/dL) among girls and young women from 50% to 40% with a power of 80%. In our previous randomized controlled trials in Tanzania we have encountered a low loss to follow up of around 5%. These were facility-based trials unlike this one where the subjects are enrolled and followed up at home. Therefore we anticipate a loss to follow up smaller than 5% and the sample size of 600 per limb covers losses of 6% of subjects during follow up.

***Interventions:***

The interventions to the three groups in the trial will consist of the following:

Intervention 1: A multivitamin and micronutrient supplement that constitutes 1 RDA of Vitamins A, B1, B2, B6, B12, niacin, C, E, and folic acid (Table 1) along with 30 mg of elemental Iron taken daily.

| Nutrient | Single RDA multivitamins |
|---|---|
| Vitamin A | 2500 IU |
| Vitamin B1 | 1.4 mg |
| Vitamin B2 | 1.4 mg |
| Vitamin B6 | 1.9 mg |
| Vitamin B12 | 2.6 μg |
| Niacin | 18 mg |
| Vitamin C | 70 mg |
| Vitamin E | 10 mg |
| Folic acid | 0.4 mg |

Intervention 2: A nutrient supplement that consists of 0.4 mg of folic acid and 30 mg of elemental iron taken daily. Placebo: Girls in the placebo group will get 0.4mg of Folic acid alone.

***Follow-up and Data Collection*:**

Enrolled girls aged 15 to 29 years will be identified during the first DSS visit at the beginning of the study and followed up every month. Each participant will receive a unique, 6-digit identification number and an identification card. A study research worker will visit them at home and record all clinical information obtained through a detailed history and examination on standardized case report forms (CRFs). The research worker will also collect bottles of micronutrient supplements handed out at previous visits and replace them with fresh bottles of supplements once every month. A structured questionnaire will be used to record responses to questions on factors that influenced or could influence compliance to the supplements and any adverse effects experienced.

***Lab samples:***

At enrollment and completion of the study, health workers will collect 10 ml of peripheral blood by phlebotomy in vaccutainers. These samples will be transported to the Rufiji DSS site, aliquoted every day and stored at -20 C. At the end of each week they will be transported to the MUHAS-Harvard collaborative Lab at MUHAS, Dar es Salaam for further processing.

The following measurements will be made at the laboratory using standard methods:

1. Hemoglobin (at enrollment and at the end of the trial)
2. Peripheral blood smear and red blood cell morphology for type of anemia.
3. Peripheral malaria parasitemia (at enrollment and at the end of the trial)

Remaining samples of blood will be frozen and stored at – 70 C for other biochemical analysis subsequently.

***Definition of endpoints:***

Primary endpoints:

Compliance: Compliance will be determined primarily by pill counts at each visit when the bottles of supplements are returned at each visit. Compliance will be calculated at

each visit using the formula: Compliance = 100*(days in a month – pills returned) / days in the month

Anemia defined as Hemoglobin < 12gm/dL at 6 months of intervention. Hemoglobin concentration will also be analyzed as a continuous outcome.

Secondary endpoints: (a) Weight gain during intervention – difference in weight between weight at randomization and weight at 6 months of intervention; (b) Mid Upper Arm circumference at end of trial; (c) Peripheral malaria parasitemia, defined as fever in the past 72 hours with any malaria parasitemia in peripheral blood.

***Data Management*:**

The questionnaires will be transferred to the project data center in Rufiji where two data entry clerks will enter them into a Microsoft Access Database. Data checks will be run using SAS software and questionnaires with wrong data will be returned to the field for correction.

***Study Participants Safety*:**

All study participants will be provided with care and treatment that adhere to national guidelines. All study personnel - qualified antenatal nurses and health workers will be rigorously trained at the beginning of the trial in standardized protocols of interaction, history taking and clinical examination and blood sampling. They will undergo regular retraining every 6 months to maintain utmost quality in providing antenatal care. All home visits will be made at the most convenient times of the day and visits will be rescheduled in case of any such request made by the participant.

A data safety and monitoring board will be formed to direct data analyses for assessing treatment effects during the trial. The committee will meet once every 6 months to assess progress of the study, and to monitor safety and efficacy of the supplements. The trial would be stopped if the committee finds early indication of significant differences between the treatment groups.

***Timeline:***

The duration of the project will be 12 months. In the first month, we will develop the research instruments and questionnaires. Enrolment and randomization will begin from the second month and will be completed over 3 months. Follow up for the last enrollee will be completed by 10 months. The final two months will be devoted to data pooling and analysis. In month 12, the results of the study and the information materials will be introduced to policy makers so that they can be made available for the entire country and possibly other countries in sub-Saharan Africa.

***Dissemination of Results and Policy Implications:***

We will meet with key decision makers to inform them on the evidence of multivitamin supplements prior to pregnancy to bring about a policy change. Subsequently, we will meet with key stakeholders to operationalize the introduction of such vitamins into care

of young non-pregnant girls and women. Meetings will be convened with Dr. Mtasiwa, the current Tanzanian Chief Medical Officer (equivalent to Director General in the U.S.) and his staff. Dr Mtasiwa has the leading role in initiating and approving health policy change in Tanzania. Concurrently, we will present the findings to the Director of Preventive Services, Dr. Mmbando. Both will subsequently liaise with the Director of Reproductive and Child Health Dr. Sanga, who is responsible for care related to pregnancy and reproductive health. With the help of these three key figures, the findings will be presented during a Ministry of Health Management Meeting that will also be attended by the Chair of the Development Partner Group, i.e. the group representing the all development agencies such as World Bank, UNICEF, and non-governmental organizations. These processes may have to be followed by separate meetings of the Ministry of Health and the Development Partner Group.

After obtaining this high-level support, we will organize and conduct a meeting with key stakeholders in nutrition and reproductive health. These will include United Nations-organizations such as the United Nations Children's Fund, World Food Programme, the World Health Organization, non-governmental organizations such as Helen Keller International and possibly Doctors without Borders, as well as government organizations such as the United States Agency of International Development and the Tanzanian Food and Nutrition Centre. The meeting will be planned and conducted in close collaboration with the Ministry of Health. This meeting will provide an opportunity to catalyze the support for multivitamins in Tanzania.
